# Impact of Pasture-Based Diets on the Untargeted Metabolomics Profile of Sarda Sheep Milk

**DOI:** 10.3390/foods12010143

**Published:** 2022-12-27

**Authors:** Gabriele Rocchetti, Pier Paolo Becchi, Lorenzo Salis, Luigi Lucini, Andrea Cabiddu

**Affiliations:** 1 Department of Animal Science, Food and Nutrition, Università Cattolica del Sacro Cuore, 29122 Piacenza, Italy; 2 Department for Sustainable Food Process, Università Cattolica del Sacro Cuore, 29122 Piacenza, Italy; 3 Agris, Agricultural Research Agency of Sardinia, Loc. Bonassai, 07040 Olmedo, Italy

**Keywords:** UHPLC-QTOF, foodomics, polyphenols, grazing dairy sheep, permanent pasture

## Abstract

In this work, untargeted metabolomics was used to shed light on the impact of different pasture-based diets on the chemical profile of Sarda sheep milk. The study considered 11 dairy sheep farms located in Sardinia, and milk samples were collected in 4 different periods, namely January, March, May, and July 2019, when all sheep had 58, 98, 138, and 178 days in milk, respectively. The animal diet composition was based on the intake of grazed herbage in natural pasture, hay, and concentrate. Overall, the combination of two comprehensive databases on food, namely the Milk Composition Database and Phenol-Explorer, allowed the putative identification of 406 metabolites, with a significant (*p* < 0.01) enrichment of several metabolite classes, namely amino acids and peptides, monosaccharides, fatty acids, phenylacetic acids, benzoic acids, cinnamic acids, and flavonoids. The multivariate statistical approach based on supervised orthogonal projections to latent structures (OPLS-DA) allowed us to predict the chemical profile of sheep milk samples as a function of the high vs no fresh herbage intake, while the prediction model was not significant when considering both hay and concentrate intake. Among the discriminant markers of the herbage intake, we found five phenolic metabolites (such as hippuric and coumaric acids), together with lutein and cresol (belonging to carotenoids and their metabolites). Additionally, a high discriminant power was outlined for lipid derivatives followed by sugars, amino acids, and peptides. Finally, a pathway analysis revealed that the herbage intake affected mainly five biochemical pathways in milk, namely galactose metabolism, phenylalanine metabolism, alpha-linolenic acid metabolism, linoleic acid metabolism, and aromatic amino acids involved in protein synthesis (namely tyrosine, phenylalanine, and tryptophan).

## 1. Introduction

European sheep milk production represents about 29% of the world production, and the Italian contribution is about 3%. Sardinia contributed 65% to the total dairy milk sheep produced in Italy, which is all processed into cheese, with about 30% belonging to the protected denomination origin (PDO). Additionally, most of the dairy sheep farms are located in the plains or in the hills (considering altitudes below 500 m). Overall, one of the most adopted farming systems is represented by the semi-extensive one, where ewes graze on natural and/or crop pastures, which represent 80% of the dry matter (DM) annually ingested by the flock. Additionally, the remaining 20% of DM consumption consists of roughages and concentrates [1]. In the last decades, intensive farming systems are being largely adopted by using great amounts of concentrate and dried fodder, with less grazing activity, thus resulting in natural pasture abandonment in the less favored areas [2,3]. It is well assumed that pasture-based diet significantly increased the contents of carotenoids, tocopherol, polyunsaturated fatty acids (PUFA), and monounsaturated fatty acids (MUFA) in dairy products compared to conserved forage-based diets, as recently reported by Cabiddu et al. [4]. Actually, results on the effect of natural pasture-based diet on milk contents are very scanty in particular considering the effect of the botanical composition and phenological stage of plants on milk composition. Overall, even if it is not clear, feeding management affects milk composition in ruminant products in a large way for milk compared to meat, decreasing when moving from cows to sheep and goats [4].

According to the literature [5], sheep milk shows a different composition when compared to cow milk as it is mainly produced by the seasonal breeding of ewes. Instead, looking at cow milk, cows are recognized to have year-round breeding. Therefore, the particular season of the year considered represents a driving factor towards the actual sheep milk composition, since from late winter to middle spring plants are usually at the vegetative stage, whereas from late spring to summer plants are in the reproductive/maturity stage. In the last years, several analytical approaches have been used to evaluate the chemical composition and metabolic profile of sheep’s milk. Among these latter approaches, metabolomics (both targeted and untargeted) has emerged as an essential tool to obtain metabolites for prediction of herd health, techno-functionality, and mainly product authentication [6]. In a previous work, Caboni et al. [7] provided a metabolomics comparison between sheep’s and goat’s milk because a rising interest in their nutritional and health-promoting properties. These authors pointed out that sheep’s milk was particularly abundant in arabitol, citric acid, α-ketoglutaric acid, glyceric acid, myo-inositol, and glycine. Moreover, a LC-QTOF/MS untargeted metabolomics approach was recently used to evaluate the impact on milk composition following the replacement of soybean hulls with cocoa husks in the ewes’ diet [8].

However, the reviewed scientific literature suggests that data correlating sheep’s milk metabolomics profile to a certain feeding system, such as the potential influence of permanent pasture on milk traits, are still scanty. Therefore, in this work, we used a comprehensive untargeted mass spectrometry approach to establish those main milk markers sourced from a pasture-based diet, such as small molecular weight compounds, secondary metabolites, and fatty acid derivatives. The final aim was to propose significant biomarkers likely useful to establish future threshold values to test the link between dairy products (i.e., milk from pasture-based diets) and the territory of origin.

## 2. Materials and Methods

### 2.1. Collection of Milk Samples and Main Characteristics of Feeding System

This study was conducted in 2019 and considered 11 dairy sheep farms located in a hilly central area of Sardinia (i.e., in the region of Màrghine (300–700 m a.s.l.). In each farm, bulk milk samples (50 mL) from morning session milking were collected on 4 sampling days over 4 months, namely January, March, May, and July, 2019, when all Sardinian sheep had 58, 98, 138, and 178 days in milk, respectively. The animals’ estimated diet composition was indicated as intake of (a) grazed herbage in natural pasture (HeI), (b) hay, and (c) concentrate. Detailed information regarding the feeding system considered for each sampling day are reported in Table 1 and Table 2. Briefly, dry matter intake at each sampling date was estimated considering milk yield and composition, animal body weight, amount of hay, and concentrate supplementation considering the time of access to pasture as also reported by [4]. Additionally, the herbage intake was estimated by the difference between the potential intake capacity of the animals at the different sampling dates and the encumbrance provided by the other feedstuffs fed to the sheep in agreement with [3]. However, more details about the management factors (e.g., feeding supplementation, pasture botanical composition, and chemical composition of diets) are reported elsewhere [4].

### 2.2. Extraction Step of Milk Metabolites from the Different Collected Samples

The extraction process for untargeted metabolomics profiling was performed as previously reported by Rocchetti et al. [9], with small modifications. Briefly, a skimming of milk samples was carried out using a centrifugation step (4500× *g* for 10 min at 4 °C). Afterwards, the 45 skim sheep milk samples were thawed and finally vortex mixed; 2 mL of each sheep milk sample was extracted with 14 mL of acetonitrile (LC-MS grade, Sigma-Aldrich, Madison, CA, USA) acidified with 1% formic acid. Following a vortexing step for 2 min, samples were finally ultrasonic processed (DU-32 ARGOLab, Milan, Italy) for 5 min at maximum power (120 Watt) to promote the extraction of metabolites. Thereafter, samples were centrifuged for 15 min (at 12,000× *g*, considering cold conditions at 4 °C) to promote the precipitation of large biomolecules (such as proteins). Finally, the supernatants were filtered using a 0.22 μm cellulose syringe filters. Samples were collected in amber vials until further instrumental analysis based on UHPLC-QTOF mass spectrometry.

### 2.3. UHPLC-QTOF Mass Spectrometry Approach to Profile Milk Metabolites

The metabolomics profiling was performed by exploiting a chromatographic system (Agilent 1200 series, from Agilent Technologies, Santa Clara, CA, USA) hyphenated with a QTOF mass spectrometer (Agilent 6550 iFunnel, from Agilent Technologies, Santa Clara, CA, USA) [9]. The sheep milk extracts were analyzed under positive polarity (ESI+), in full scan mode, considering an m/z range of 100–1200. Additionally, the acquisition rate consisted of 0.8 spectra/s, considering an extended dynamic range mode (nominal mass resolution = 30,000 FWHM). The chromatographic gradient consisted of the exploitation of water/acetonitrile (both LC-MS grade, from Sigma-Aldrich, Milan, Italy) moving from 6% up to 94% acetonitrile in 35 min. Furthermore, 0.1% formic acid was used as phase modifier and added to both water and acetonitrile. The Agilent Zorbax Eclipse Plus C18 column (50 × 2.1 mm, 1.8 μm) was selected for the chromatographic separation. Regarding the electrospray conditions followed, they are reported elsewhere [10]. The injection volume was 6 μL (*n* = 3), and the sequence injection was randomized to avoid bias during the sample running. Additionally, quality control samples (QCs) were prepared by pooling a small aliquot of extract from each milk sample (20 μL). These QCs were analyzed adopting a typical data-dependent MS/MS mode, selecting 12 precursor ions per cycle (1 Hz, 50–1200 m/z, positive polarity, active exclusion after 2 spectra), and considering typical collision energies (i.e., 10, 20, and 40 eV), using the software MS-Dial and exploiting the information reported on the database MoNA (Mass Bank of North America, https://massbank.us/, accessed on 13 September 2022). Additionally, a representative chromatogram (TIC) of the pooled QC sample together with those metabolites confirmed by MSMS can be found in Table S1.

Following the instrumental analysis, the software Agilent Profinder B.06 (Agilent Technologies) was used for the data elaboration and identifications step. The identification was carried out considering several parameters, such as the monoisotopic mass information, the isotopic profile (both spacing and ratio) of each milk metabolite, and the selection of a 5 ppm tolerance for a better mass accuracy achievement. Therefore, under our experimental conditions, a Level 2 of identification was achieved, according to the standard conditions reported by COSMOS Metabolomics Standards Initiative [11]. The mass features were identified against two comprehensive databases, namely Milk Composition Database (MCDB) [12] and the database Phenol-Explorer 3.6 (http://phenol-explorer.eu/, accessed on 13 September 2022). The databases used allowed us to consider the most important milk metabolites to study the impact of different feeding conditions. Regarding data pre-processing and data filtering, we used the software Agilent Profinder B.06; overall, compounds were retained when passing mass accuracy (5 ppm) and frequency of detection (within 100% of replications in at least one sample grouping) thresholds. Therefore, as reported in a previous work by Foroutan et al. [12], we used the term “metabolite species” referring to milk metabolites with non-unique chemical formulas or masses (e.g., lipids and their isomeric structures). Finally, the term “unique compound structures” was used to refer to those compounds having a unique chemical formula or mass.

### 2.4. Multivariate Statistical Data Analysis

The multivariate statistical data analysis was carried out exploiting two different software applications, namely MetaboAnalyst 5.0 [13] and SIMCA 13 (Umetrics, Malmo, Sweden). The step-by-step workflow of multivariate statistical data analysis is reported in detail in our previous work [14]. Overall, following a data normalization step, we used supervised multivariate statistics based on orthogonal projections to latent structures discriminant analysis (OPLS-DA) to evaluate the differences and analogies induced by the feeding systems under investigation. To this aim, the discriminant power of each milk metabolite annotated was evaluated through the application of a variable selection method, also known as VIP (i.e., variables importance in projection), selecting a typical cut-off range for discrimination (VIP score) higher than 0.8. Additionally, the accumulation trend of those discriminant VIP compounds was evaluated through the calculation of fold-change values based on fold-change analysis (FC, cut-off value = 2) and the analysis of variance (ANOVA, *p* < 0.05; post hoc test: Tukey HSD; multiple testing correction: Bonferroni FWER). In addition, the software MetaboAnalyst 5.0 was used to perform an enrichment analysis that was used to inspect metabolomics-dataset in order to check what chemical classes were actually the most represented within the features annotated. Finally, the most significant metabolic pathways underlined by VIP discriminant metabolites were evaluated by using the online software MetaboAnalyst (using a specific pathway library: Kyoto Encyclopedia of Genes and Genomes, KEGG, https://www.genome.jp/kegg/, accessed on 13 September 2022).

## 3. Results and Discussion

### 3.1. Untargeted Screening of Sheep Milk Samples by UHPLC-QTOF-MS

**Figure 1:**
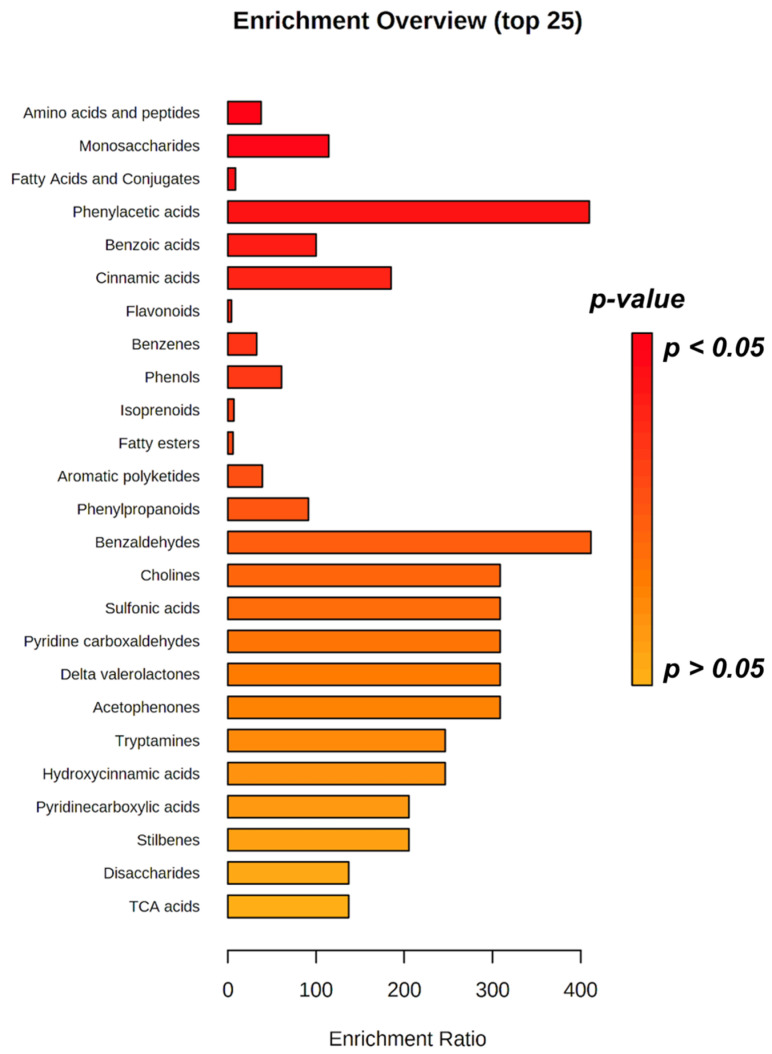
Enriched chemical classes considering sheep milk metabolites annotated by untargeted UHPLC-ESI-QTOF-MS. Abbreviations: tricarboxylic acids (TCA).

In this study, the untargeted screening based on UHPLC-QTOF mass spectrometric analysis of ewes’ milk allowed us to identify 406 compounds according to a level 2 of confidence in annotation. A comprehensive list of all the metabolites is reported in Table S1, together with their individual raw abundance values and mass spectra collected for each sample. Additionally, a representative chromatogram (TIC) of the pooled QC sample together with those metabolites confirmed by MSMS against the database MoNA can be found in Table S1. Among those metabolites confirmed by the MSMS strategy, we found 19 compounds mainly belonging to amino acids (such as tyrosine), polyphenols (such as hippuric acid and other phase-II metabolites), fatty acid derivatives (such as 2-methylbutyroylcarnitine), and nucleobases (such as uracil and guanine) (Table S1). As the next step, an enrichment analysis was performed to evaluate the most represented classes of metabolites in the metabolomics dataset. The output obtained of is reported as Figure 1.

As shown in the figure, the most significantly enriched class of metabolites belonged to amino acids and peptides, followed by monosaccharides, fatty acids and conjugates, phenylacetic acids, benzoic acids, cinnamic acids, and flavonoids. The wide number of features annotated using our metabolomics workflow is consistent with the overall complexity of the food matrix (i.e., milk) under investigation. The chemical composition of sheep milk can be significantly affected by numerous production factors representing the farming system, such as genotype, method of milking, stage and number of lactations, and farming methods (including the feeding system followed by ewes) [15,16]. Actually, the link between all of these aspects can be really difficult and complex to fully evaluate [17], but among all the factors, the feeding and seasonal breeding of ewes play a major role in modulating the chemical composition of milk. Haenlein and Wendorff [18] reported that the chemical composition of sheep milk is largely affected by seasonal breeding of ewes. Therefore, the season of the year represents for sure a driving factor towards the definition of the actual sheep milk composition. According to the literature, sheep milk contains 18.3% total solids, 6.0% fat, 12.3% non-fat solids, 4.9% lactose, 0.94% ash, and 5.2% protein [5]. Interestingly, characteristic of sheep milk are the higher concentrations of butyric acid, conjugated linoleic acid (CLA), and omega-3 fatty acid content when compared with other ruminant milks [19]. Considering the difference in terms of fatty acid profile, the effects of feeding management [20] and breeding [21] are well recognized. In particular, pasture-based diets increase PUFA contents such as linoleic and linolenic acids in cow and small ruminant milk products. The presence of ω-3 and ω-6 FA in milk fat together with other less common FA (such as linoleic acid isomers), is gaining a great interest due to the consumer demand for a healthy diet [22]. However, it is important to consider that a greatest seasonal difference can be measured in sheep milk CLA, e.g., 1.28% in summer and 0.54% at the end of the winter period [5]. In addition, season affects milk fatty acid profiles as a consequence of forage species and their phenological stage, as previously reported by Cabiddu et al. [22]; in fact, regarding the milk CLA content in sheep milk, a more significant effect was found in legumes than in grass when plants turn from the vegetative (winter period) to the reproductive stage (late spring period). Therefore, looking at the chemical composition revealed by untargeted metabolomics (Table S1), our findings generally agree with those reported in the scientific literature, highlighting a great abundance of linoleic acid isomers, amino acids, and peptides of functional interest. In this regard, Barłowska et al. [23] showed that goat and sheep milk are characterized by the best composition of exogenous amino acids. Therefore, these milk samples fully cover the requirement for all essential amino acids. Accordingly, in this work, we successfully annotated several essential amino acids, such as valine, leucine, isoleucine, methionine, and phenylalanine (Table S1). Another important factor that has a crucial role on milk composition is represented by the inclusion of phenolic compounds in the diet. Overall, the transfer of these compounds in animal tissues increases the quality of livestock products and potentially improves the oxidative stability of milk and cheese [24]. As products of plant secondary metabolism, polyphenols can naturally occur in the diet of herbivorous farm animals. In this regard, a higher content of phenolics has been previously detected in sheep milk as a consequence of the administration of a pasture-based diet compared with concentrate- and silage-based diets [25]. Dietary polyphenols can affect milk fatty acid profiles, reducing the growth of some strains involved in the biohydrogenation of polyunsaturated fatty acids when considering sheep ruminal metabolism [26]. Additionally, as extensively reviewed by Zhu et al. [27], relatively few comprehensive metabolomics studies on milk from smaller ruminants, such as goats and sheep, have been published so far; therefore, our findings support the utilization of untargeted metabolomics to shed light on the chemical differences triggered by different typical ewe feeding regimens. The level of such bioactive compounds detected could be explained by the content of legumes and forbs plants species in the pasture, which is also in agreement with Mariaca et al. [28].

### 3.2. Discrimination of Milk Samples as a Function of the Different Feeding Strategies (Herbage, Hay, and Concentrate Intake)

To investigate the correlation between milk metabolomics profile and animal diet composition, a multivariate supervised orthogonal projection to latent structures discriminant analysis (OPLS-DA) was carried out considering as class discriminant parameters the different feeding systems, i.e., based on (a) herbage intake, (b) hay intake, and (c) concentrate intake. Firstly, multivariate statistics based on supervised OPLS-DA prediction models were used to evaluate the impact of hay- and concentrate-based feeding strategies on the metabolomics profile of sheep milk. However, as reported in Table S1, although a separation trend could be observed, both models were characterized by a non-significant prediction ability (Q^2^ < 0.5). These results agree with a previous paper which considered the effect of hay or concentrate supplementation on macro- and micro-components in milk [4]. Therefore, considering the scarce prediction ability, no discriminant metabolites were extrapolated from either model.

**Figure 2:**
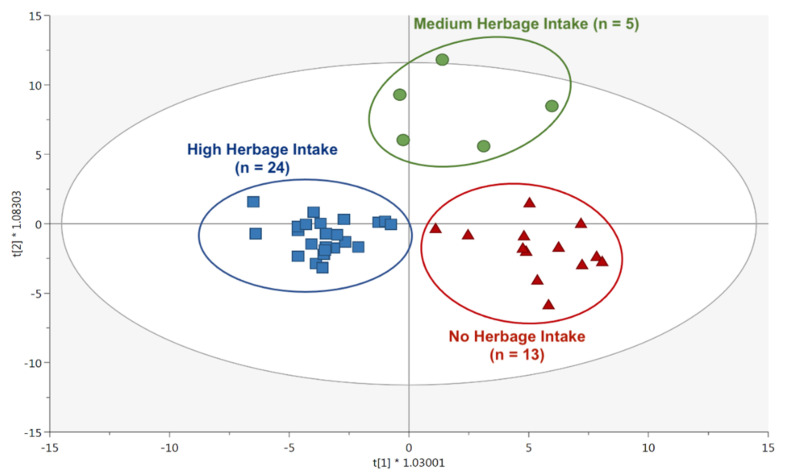
OPLS-DA score plot on the metabolomics profile of Sarda sheep milk considering the different fresh herbage intake levels as discriminant parameter.

As the next step, we focused attention on the ability of the untargeted metabolomics-based approach to provide potential biomarker compounds able to highlight the effect of pasture forages on the sheep milk metabolome. In particular, the milk samples were classified according to the average contribution of fresh herbage in the daily diet, being high (High-HeI), medium (Medium-HeI), and no-herbage intake (No-HeI). As can be observed from the OPLS-DA score plot in Figure 2, the orthogonal latent vector was effective in highlighting the effect of herbage intake on the milk metabolomics profile, thus providing a clear grouping and separation trend related to the classification criterion chosen. Additionally, this OPLS-DA model showed high goodness of fit (R^2^Y (cum) = 0.806) and prediction ability (Q^2^ = 0.510).

Then, to provide the most discriminant compounds related to the herbage intake (HeI), the variable importance in projection (VIP) method was associated with a fold-change (FC) analysis for the comparison “High-HeI vs. No-HeI”. This comparison resulted more informative about the potential impact of pasture-based diets on the chemical profile of sheep milk, establishing the main biomarkers. Overall, 37 compounds were found to possess the highest discriminant potential, having a VIP score value >1 and these markers are reported in Table 3, grouped in chemical classes (according to the classification reported on the comprehensive Milk Composition Database). Additionally, one-way ANOVA showed *p*-values < 0.05 for 24 out of 37 compounds (Table 3). From the VIP analysis, we found that a diet rich in herbage affected the levels of several compounds, mainly ascribed to lipids, sugars, and other lower-molecular-weight derivatives. Interestingly, the lipids detected were characterized by a general up-accumulation when considering the High-HeI vs. No-HeI comparison, thus indicating that the pasture-based diet had a particular incidence in the fatty acid profile of ewes’ milk. In particular, the lipid compounds that were found to be associated with high LogFC score values were mainly diacylglycerols and polyunsaturated fatty acids (PUFA). These results agree with the reviewed scientific literature [18], highlighting the positive effect of pasture-based diets on the milk PUFA content because of the higher content of fatty acid precursors in fresh herbage [29]. An important role in increasing milk PUFA content comes from plant secondary metabolites, which occur in greater number compared to crop pasture. In Table 1, linolenic acid and polyphenols appear as discriminant markers between pasture-based diets and the conserved forage group, thus confirming our previous results found at the farm level [4].

In our experimental conditions, we found two main discriminant PUFA compounds, such as linolenic acid and stearidonic acid. Since linolenic acid is the main fatty acid which occurs in fresh herbage (about 80% of total fatty acids) and which is mainly biohydrogenated by ruminal bacteria to biohydrogenated intermediates such as CLA and stearic acid [29]. However, fractions of these molecules are detected in ewe tissues and milk, reflecting their enrichment in the ruminant’s diet. In this regard, pasture supplementation of animal diet has been reported as a sustainable dietary strategy able to increase the content of n-3 fatty acids, with positive effects on human health [30]. Other important accumulation trends were pointed out considering the amino acid composition detected by the VIP analysis. Amino acids showed a high average LogFC value (i.e., average LogFC value = 4.92), mainly characterizing pasture-based ewes’ milk samples. These results were consistent to those reported by other studies [31,32], which found more products of amino acid metabolism in the resultant milk produced by herbage-based diets characterized by a high protein-to-digestible-carbohydrate ratio. As concerns the discriminant VIP compounds, phenylalanine, L-tyrosine, the dimer proline–isoleucine, and taurine showed strong up-accumulation trends. Tyrosine has already been detected [33] as biomarker of pasture-derived milk, and it is also recognized as one of the most abundant free amino acids in raw sheep milk [34]. Regarding the other amino acid compounds, it is complicated to find stronger correlations with the feeding regimen followed by the ewes. For example, taurine, highlighted for its evident up-regulation, derived directly from sulfur-amino acid derivatives and has important function in humans as an important nutrient for the newborn [35]. All these results partially agree with Cabiddu et al. [36], who found a small relationship between milk total phytanic content and daily fresh herbage contribution to daily diet intake.

Moreover, pasture-based diets also have a greater influence on phenolic composition than concentrate and conserved forage-based diets. Our findings revealed the presence of important phenolic acids and carotenoids such as hippuric acid, coumaric acid, lutein, and cresol in milk as a function of pasture-enriched diets. As previously observed [4,37], feeding livestock with pasture-based systems enhances the oxidative stability of ewes ‘milk with higher levels of lutein and other milk carotenoids (with respect to conserved forage-based diets) according to the high levels of carotenoids in fresh herbage [38]. In addition, *p*-cresol appears a crucial metabolite related to milk flavor attributes, and it is produced from the rumen metabolism of isoflavones derived from pastures. Interestingly, a previous work by Faulker et al. [39] indicated *p*-cresol (a degradation product of β-carotene) as a potential biomarker for bovine milk derived from pasture. These authors also associated *p*-cresol with the deamination and decarboxylation of certain amino acids, such as tryptophan and tyrosine, due to higher levels of available protein in the forage-based diet. Therefore, our findings also support the utilization of *p*-cresol as discriminant marker of the pasture-based regimen when considering sheep milk. It is of interest that *p*-cresol is also an intermediate of polyphenol oxidase (PPO) activity, and, as reported by Cabiddu et al. [40], the high biodiversity rate of pasture in Sardinia is also characterized by high levels of PPO activity, so probably the reason for an increase in milk *p*-cresol could be due to the combination effect of PPO and lutein content in fresh forages. Additionally, the high levels detected for hippuric acid (LogFC = 1.30) agree with a higher herbage intake, considering that this phenolic metabolite (derived from a conjugation reaction between benzoic acid and glycine) has been widely detected in different animal biofluids and related to pasture-based feeding regimens [41].

### 3.3. Pathway Analysis of Sheep Milk Samples According to the Herbage Intake

**Figure 3:**
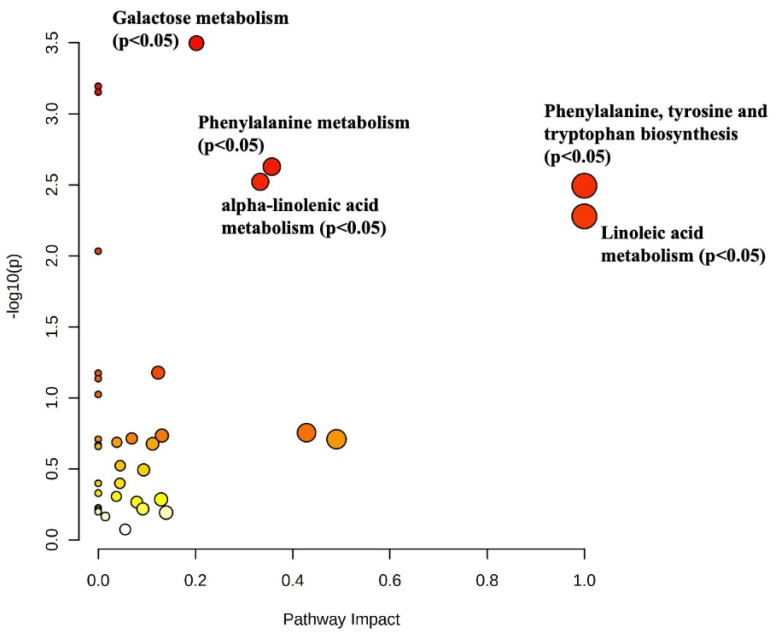
Metabolic pathway analysis built according to the discriminant and significant metabolites identified through VIP analysis in milk for the comparison High-HeI vs. Low-HeI samples. The x-axis describes the pathway impact, while the y-axis represents the relative pathway enrichment.

To confirm the impact of the herbage intake on the milk metabolome, a pathway analysis was built considering the VIP-discriminating metabolites of the comparison *High-HeI* vs. No-HeI samples, and the obtained output is reported in Figure 3. As a general consideration, several metabolic pathways were highlighted as extremely significant and impacting at metabolomics level, involving mainly galactose metabolism, linoleic acid metabolism, and phenylalanine, tyrosine, and tryptophan metabolism. Interestingly, galactose metabolism was annotated with a huge Log *p*-value (i.e., 3.5), likely affected by the presence of galactitol, a breakdown product of lactose degradation. In our experimental conditions, both galactitol and lactose showed up-accumulation trends, and a recent work by Angeles-Hernandez et al. [42] revealed that high-concentrate-based diets are richer in lactose than those fed with high-forage strategy. In addition, linoleic acid and alpha-linoleic acid metabolism showed a crucial effect on the discrimination of sheep milk samples according to the different level of herbage intake. As already reported in this work, grazing has an important impact on n-3 PUFA content, especially on the high distribution of alpha-linolenic acid. Interestingly, Jin et al. [43] showed that concentrate-based diets were characterized by a large amount of linoleic acid derivatives and their presence has been proposed as possible marker of indoor feeding system. In addition, Lock et al. [44] demonstrated the efficacy of conjugated linoleic acid (CLA) supplements in dairy cow diets as potent inhibitors of milk fat synthesis. Looking at our findings, milk samples with a low herbage intake were characterized by a lower amount of PUFA and glycerophospholipids when compared with milk samples from higher grass-based intakes. This trend partially agreed with previous findings reported in literature for cow’s milk; however, further studies are needed to better evaluate the impact of linoleic acid metabolism on lipid synthesis in sheep rumen. Regarding the other affected metabolomics pathways, the up-accumulation of certain amino-acid-related biosynthetic routes, such as those involving tyrosine and tryptophan, are consistent with the increase in some discriminant VIP marker compounds, such as the previously cited *p*-cresol (a VIP marker of the herbage intake) and to the higher protein levels associated with forage-based diets.

Overall, this untargeted metabolomics-based approach demonstrated a great ability to investigate possible correlations between milk chemical composition and sheep feeding regimens based on permanent grasslands. Our findings revealed the influence of grazing herbage intake on fatty acid composition but also in the amino acid and polyphenolic profiles. Milk from grazing sheep has a specific influence on n-3 PUFA content associated with other bioactive properties, such as carotenoids and polyphenols.

## 4. Conclusions

A comprehensive UHPLC-ESI-QTOF allowed us to highlight the impact of different pasture-based diets on the chemical profile of Sarda sheep milk, considering animal diets based on the intake of grazed herbage in natural pasture, hay, and concentrate. Several comprehensive databases on food, such as the Milk Composition Database and Phenol-Explorer, allowed the identification of 406 metabolites, with amino acids and peptides, monosaccharides, fatty acids, phenylacetic acids, benzoic acids, cinnamic acids, and flavonoids being the most significantly enriched. A multivariate statistical approach based on supervised OPLS-DA successfully predict the chemical profile of sheep milk as function of the high vs. no fresh herbage intake, while recording a non-significant prediction when considering both hay and concentrate intake. The most discriminant marker compounds of the herbage intake were 5 phenolic metabolites, followed by carotenoids. In addition, lipid derivatives followed by sugars, amino acids, and peptides showed a great discrimination potential. Finally, five biochemical pathways in milk were found to be affected by the inclusion of fresh herbage, such as galactose metabolism, phenylalanine metabolism, alpha-linolenic acid metabolism, linoleic acid metabolism, and aromatic amino acids (tyrosine, phenylalanine, and tryptophan). Taken together, our findings highlight the suitability of untargeted metabolomics as powerful tool in milk authenticity studies, thus supporting what is stated by similar studies already available in the scientific literature [45,46]. These results also need to be implemented with a large database in the next future.

## Figures and Tables

**Table 1:** Chemical composition of pastures and supplements used by farmers in the 4 sampling days.

	Pasture					Hay		Concentrate		
	Jan	March	May	July	SEM	Hay	SEM	Conc HCP	Conc. Starch	SEM
DM (%)	18.65	19.81	22.63	67.11	3.30	83.13	1.38	88.73	87.21	0.40
Ash (%DM)	12.02	12.62	9.59	7.66	0.48	7.89	0.40	5.46	3.00	0.52
EE (%DM)	3.12	2.92	3.09	2.43	0.15	1.92	0.07	3.19	2.94	0.29
CP (%DM)	16.69	16.49	13.16	5.96	0.95	8.41	0.81	15.57	9.05	0.67
NDF (%DM)	52.36	43.77	48.24	68.18	1.93	65.35	1.42	24.73	15.71	2.15
ADF (%DM)	27.07	21.51	26.51	38.93	1.25	40.42	0.80	10.74	6.00	1.10
ADL (%DM)	3.58	2.14	2.63	4.88	0.27	6.19	0.34	1.34	0.51	0.18
TPC (%DM)	1.18	1.65	1.99	1.22	0.11	0.00	0.00	0.00	0.00	0.00
Starch (%DM)	-	-	-	-	0.00	0.00	0.00	38.36	55.2	3.56

DM = dry matter; EE = ether extract; CP = crude protein; NDF = neutral detergent fiber; ADF = acid detergent fiber; ADL = acid detergent lignin; Conc. HCP = concentrate high in crude protein content; Conc. Starch = concentrate high starch content; TPC = total phenolic content (as gallic acid equivalent). SEM = standard error of means.

**Table 2:** Average contribution (%) of different feed types (HeI = fresh herbage intake, Conc. = concentrate) to the flocks’ diet throughout the milk production season, considering 11 commercial Sarda ewe farms.

Sampling	HeI	Conc.	Hay	Standing Hay
January	44.32	31.08	24.85	0.00
March	67.66	22.28	13.68	0.00
May	85.32	16.80	0.98	0.00
July	0.00	19.58	2.97	77.44

**Table 3:** VIP discriminant compounds (both down- and up-accumulated) in milk samples as a function of the fresh herbage intake (HeI vs. no-HeI). * = at least 2 isomeric structures (i.e., metabolite species). FC = fold change.

Class	Discriminant Marker (OPLS-DA)	VIP Score (OPLS-DA)	Log_2_FC (HeI vs. No-HeI)	*p*-Value (ANOVA)
Lipids and derivatives	20:5 Cholesteryl ester	1.37 ± 1.06	−3.28	0.0189
	Stearidonic acid	1.10 ± 0.48	2.32	*p* > 0.05
	TG(4:0/15:0/16:0)	1.47 ± 0.95	1.65	0.0136
	Alpha-Linolenic acid *	1.43 ± 1.13	2.42	*p* > 0.05
	TG(4:0/10:0/14:0)	1.21 ± 0.36	4.86	*p* > 0.05
	DG(12:0/20:2(11Z,14Z)/0:0) *	1.13 ± 1.02	11.71	0.0252
	DG(12:0/20:1(11Z)/0:0) *	1.19 ± 0.52	12.16	0.0389
	DG(14:0/22:5(7Z,10Z,13Z,16Z,19Z)/0:0)	1.48 ± 1.08	12.84	0.0385
	Hydroxybutyrylcarnitine	1.24 ± 0.80	13.19	0.0282
	Vitamin K1	1.65 ± 0.67	14.88	0.0028
	DG(14:0/20:1(11Z)/0:0) *	1.23 ± 0.99	15.01	0.0476
			LogFC (avg) = 7.98	
Aminoacids and derivatives	N-hydroxy-L-tyrosine	1.65 ± 1.02	−1.30	*p* > 0.05
	Arg-Thr-Lys-Arg	1.34 ± 0.89	−1.20	0.0297
	2-Aminooctanoic acid	1.22 ± 1.09	−0.75	*p* > 0.05
	2,4-Diaminobutyric acid	1.52 ± 1.18	−0.52	*p* > 0.05
	Taurine	1.07 ± 0.63	9.15	*p* > 0.05
	L-Tyrosine	1.31 ± 0.31	10.76	0.0095
	Pro-Ile	1.06 ± 0.62	11.13	*p* > 0.05
	L-Phenylalanine	1.67 ± 1.11	12.07	0.0191
			LogFC (avg) = 4.92	
Sugars and derivatives	D-Glucose *	1.63 ± 0.97	−1.62	*p* > 0.05
	Threonic acid	1.76 ± 0.91	−0.94	0.0013
	Glyceric acid	1.23 ± 0.95	−0.47	0.0136
	Alpha-lactose *	1.46 ± 0.79	0.41	0.0266
	Galactitol *	1.17 ± 0.93	11.69	0.0201
			LogFC (avg) = 1.81	
Phenolics and metabolites	4-aminobenzoic acid	1.83 ± 1.23	−0.85	*p* > 0.05
	Hippuric acid	1.69 ± 0.96	1.30	*p* > 0.05
	5-(3′,5′-dihydroxyphenyl)- gamma-valerolactone 3-*O*-glucuronide	1.36 ± 0.77	9.60	0.0292
	Coumaric acid *	1.41 ± 0.81	10.33	*p* > 0.050
	3,4,5,4′-Tetramethoxystilbene	1.16 ± 1.01	16.24	0.0498
			LogFC (avg) = 7.32	
Carotenoids and metabolites	Lutein *	1.59 ± 0.95	13.19	0.0088
	Cresol *	1.59 ± 0.61	2.23	0.0227
			LogFC (avg) = 7.71	
Other compounds	3-Methylfuran	1.45 ± 0.84	−1.61	*p* > 0.05
	Hypoxanthine	1.72 ± 0.82	−0.93	0.0059
	Inosine	1.80 ± 0.57	−0.76	0.0010
	Uracil	1.93 ± 0.84	2.76	0.0006
	Benzyl methyl sulfide	1.10 ± 1.05	4.58	0.0427
	5-Methoxyindoleacetate	1.86 ± 0.77	11.59	0.0058
			LogFC (avg) = 2.60	

## Data Availability

Data is contained within the article and Supplementary Materials.

## Supplementary Materials

The following supporting information can be downloaded at: https://www.mdpi.com/article/10.3390/foods12010143/s1, Table S1: Annotated metabolites by using an untargeted UHPLC-ESI-QTOF mass spectrometry approach considering the different sheep milk samples under investigation. Each metabolite is reported with the corresponding abundance values and mass spectrum.
